# Construction Notes

**Published:** 1895-09-21

**Authors:** 


### CONSTRUCTION NOTES.

### Extension of the Ulster Eye, Ear, and Throat Hospital.

The plans for the extension and improvement of the hospital, as finally settled, have been submitted by Mr. Seaver and approved of. The expenditure will be about £1,300, the whole of which sum is already in hand. The extension consists of two separate parts, one of which must be completed before the commencement of the other. It has been unanimously resolved that the first part should be immediately commenced. One of the rooms in the extension will be prepared specially as an operating-room for delicate operations, such as cataract, &c., and its floors and walls will be of such a character as to afford the most perfect protection from germs. The additional cost for this purpose amounts to about £65. The extension will comprise, amongst other things, four bathrooms and closets, two day-rooms for male and female patients, improved accommodation for nurses and servants, additional ward accommodation, the special operation-room, and a laundry. It is hoped that the extension will be completed in January.

